# A Case Report of Cecal Perforation Due to Sigmoid Tumor Incarceration in Inguinal Hernia

**DOI:** 10.1002/ccr3.73661

**Published:** 2026-09-28

**Authors:** Mojtaba Ahmadinejad, Armin Tajik, Hedieh Soltani, Hadi Maleki, Javad Zebarjadi Bagherpour

**Affiliations:** ^1^ Department of General Surgery Alborz University of Medical Sciences Karaj Iran; ^2^ Student Research Committee, School of Medicine Alborz University of Medical Sciences Karaj Iran; ^3^ Department of Urology, Faculty of Medicine Alborz University of Medical Sciences Karaj Iran

**Keywords:** case report, cecal perforation, inguinal hernia, sigmoid tumor, strangulation

## Abstract

This case highlights an unusual presentation in which incarceration of a sigmoid tumor within an inguinal hernia sac resulted in colonic obstruction and subsequent cecal perforation, emphasizing the importance of prompt imaging and surgical evaluation in patients with this rare combination.

## 1 Introduction

Inguinal hernia is a common surgical condition in men and adults older than 45 years, with a worldwide incidence of about 7.7% [1]. Bowel loops, omentum, and bladder are the most commonly involved organs within the inguinal hernia. An inguinal hernia is often asymptomatic; when symptomatic, it usually presents as a palpable or visible groin mass on the anterior surface of the trunk; although, in some cases, it can be complicated [2]. These complications include obstruction of intestinal loops in the sac, strangulation, incarceration, and in more prolonged cases, perforation of the strangulated intestinal wall inside the hernia and subsequent local peritonitis.

Neoplasms in the hernial sac are divided into saccular, intrasaccular, or extrasaccular based on location and origin [3]. Intrasaccular tumors originate from the organs inside the abdomen, mostly from the left colon [4]. The simultaneous occurrence of a colorectal tumor inside the sac of an inguinal hernia is a rare condition with a prevalence of 0.5%.

The main causes of obstruction in the large intestine in adults are often due to inflammatory processes (Crohn's disease and ulcerative colitis) and tumors [5]. As the obstruction progresses, the pressure inside the colon's lumen spreads backward, and a functioning ileocecal valve causes significant expansion of the cecum. According to a series of features of the cecum that will be discussed in the discussion section, the cecum is more susceptible to damage than the other parts of the colon. Finally, with perforation of the cecum, fecal peritonitis will develop, which is a fatal complication.

We present a case of perforation of the cecum following colonic obstruction caused by sigmoid tumor incarceration into the inguinal hernia sac and also conducted in accordance with the SCARE checklist [6].

## 2 Case Presentation/Examination

A 73‐year‐old Iranian male patient was admitted with a complaint of sudden and severe abdominal pain for several hours and obstructive symptoms, including vomiting and failure to defecate for 3 days. He had a history of a reducible inguinal hernia on the left side. The patient had no smoking history, and familial and personal medical history were unremarkable.

## 3 Methods

On the physical examination, tachycardia (105 bpm) and fever (38.5°C) were significant. Other vital signs were stable, and the patient was normotensive. On abdominal examination, a bulge was clearly visible in the left inguinal region, which was irreducible upon manual examination. On palpation, the abdomen had severe tenderness with rebound tenderness.

In laboratory tests, he had leukocytosis (WBC = 14,900 with 85% PMN), and normal hemoglobin (13.9 g/dL) and platelets (450,000/μL). Coagulopathy profile (PT, PTT, INR) showed no abnormalities. Findings in the performed abdominal CT scan, an incarcerated sigmoid colon containing a malignant tumor within the left inguinal hernia sac were noticeable (Figure 1).

**FIGURE 1:**
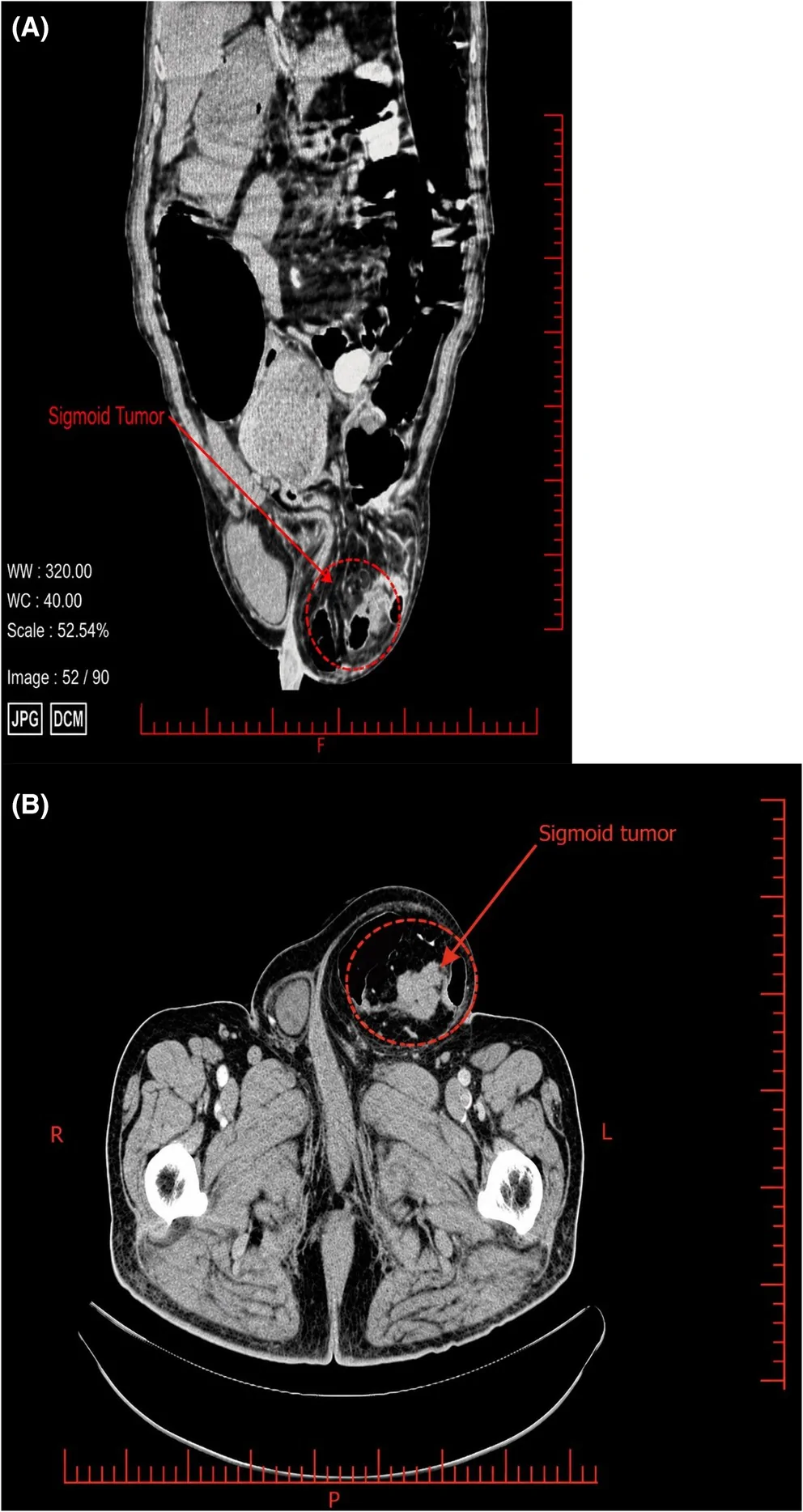
Contrast‐enhanced CT images demonstrating an incarcerated sigmoid colon containing a malignant tumor within the left inguinal hernia sac. (A) Axial CT image showing the sigmoid tumor (arrow) within the hernia sac (circled), associated with significant pneumoperitoneum. (B) Coronal reconstruction confirming the extension of the tumor‐bearing sigmoid colon into the left inguinal hernia sac (arrow and circle), associated with significant pneumoperitoneum.

Due to the presence of the generalized peritonitis symptoms, inguinal hernia reduction was not attempted, and after obtaining a peripheral blood sample and fluid resuscitation, the patient was transferred to the operating room. In the operating room, the patient underwent an emergency midline laparotomy. During exploration, a perforation was observed in the cecum (Figure 2). There was a mass in the sigmoid, which was strangulated inside the left inguinal hernia sac.

**FIGURE 2:**
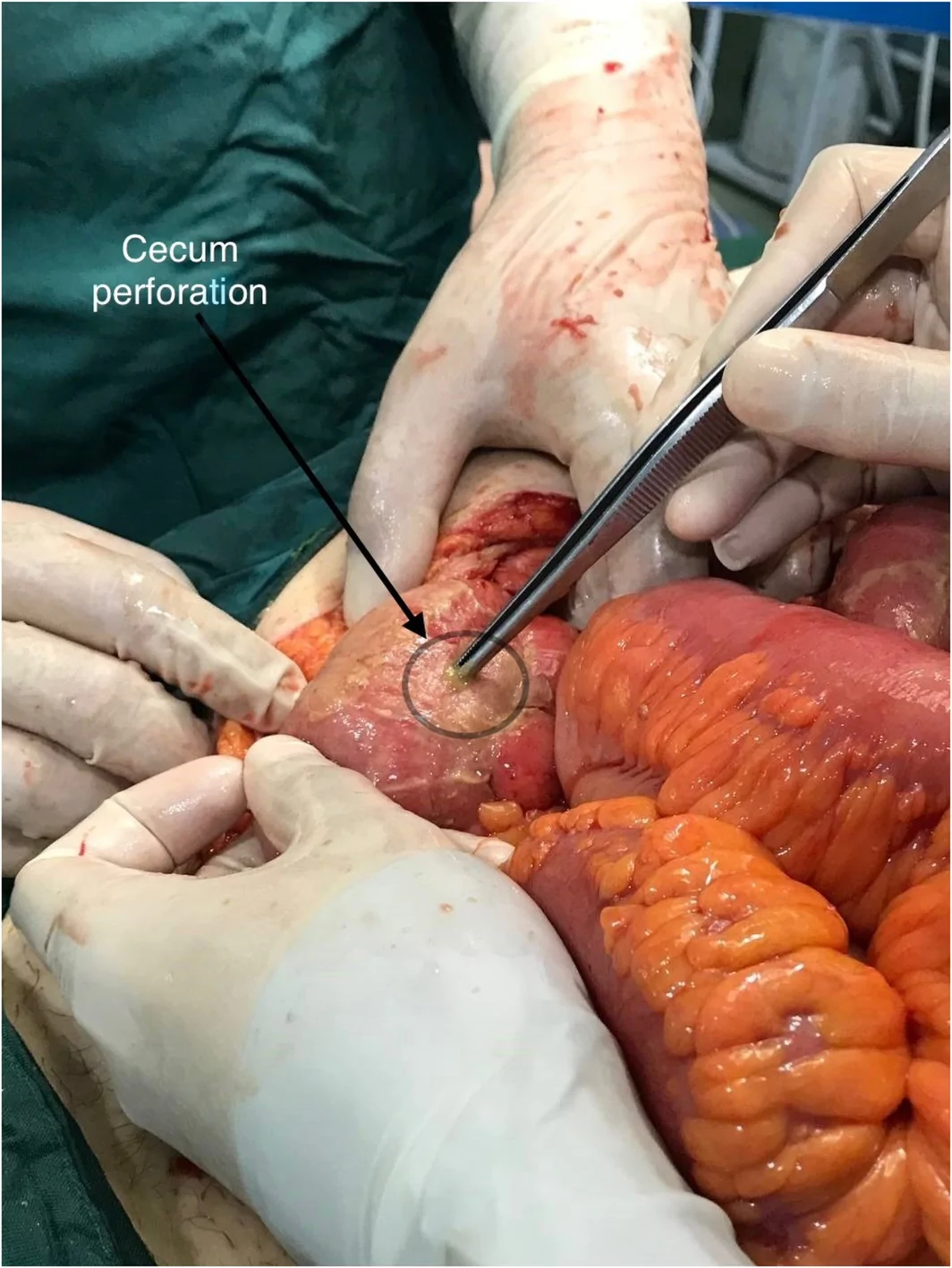
Cecal perforation during exploration.

## 4 Conclusion and Results

According to the findings, the patient underwent total colectomy and end ileostomy, and the rectal stump was closed by the Hartmann method. High ligation of the inferior mesenteric vascular pedicle was performed as part of an oncologic resection strategy. This approach was chosen to facilitate adequate lymph node dissection and ensure adherence to standard principles of colorectal cancer surgery. The patient was transferred to the surgical intensive care unit. The postoperative course was uneventful, and the patient was discharged after 5 days in good condition with stable vital signs.

In the pathology examination, an adenocarcinoma tumor was reported, and 2 lymph nodes out of 15 resected nodes were involved (T3N1). The patient underwent a metastatic workup, which fortunately did not detect distant metastases. Chemotherapy was started for the patient. After completing the course of chemotherapy and ensuring the absence of metastasis, the patient's ileostomy was closed. The patient was followed up, and no evidence of recurrence or postoperative complications was found (Figure 3). The patient was satisfied with the surgical and recovery outcome and reported significant improvement in symptoms during follow‐up.

**FIGURE 3:**
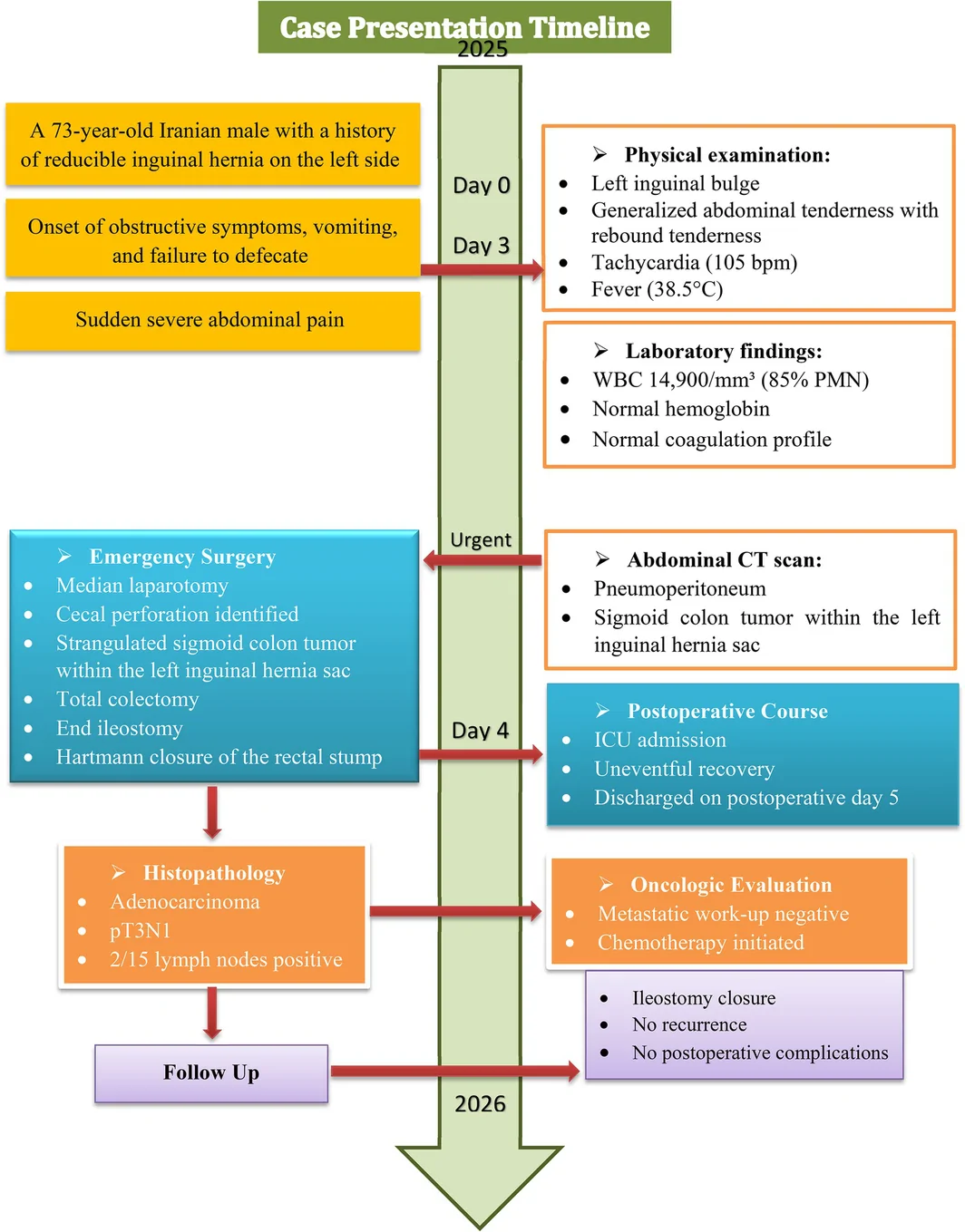
Case report timeline. Presented according to CARE guidelines.

## 5 Discussion

Inguinal hernia occurs due to abdominal wall defects and often contains bowel loops, omentum, bladder, and female reproductive system [7]. The prevalence of inguinal hernia is approximately 4000 in every 100,000 individuals older than 45 years of age worldwide, and is about 27% in males and 3% in females [8, 9]. The frequency of the inguinal hernia occurrence increases from 0.25% in 18‐year‐old patients to 4.2% in patients older than 75 years old.

An inguinal hernia is diagnosed through history taking and physical examination. Radiologic modalities, including ultrasonography (US), computed tomography (CT), and magnetic resonance imaging (MRI), should be considered to clarify the diagnosis and, more importantly, to reveal complications such as strangulation, incarceration, peritonitis, or bowel obstruction.

Generally, a neoplastic mass inside or near the hernia sac can be divided into saccular, extrasaccular, and intrasaccular. Intrasacular neoplasms originate from adjacent organs, often the sigmoid colon, and herniate into the sac. This is rare and presents in 1 in every 200 cases of colorectal cancer [10]. The sigmoid is involved more than other adjacent viscera [4].

Perforation caused by colon cancer is rare and mostly caused by lumen obstruction and increased intraluminal pressure; however, occasionally tumor cell infiltration also weakens the wall and causes perforation [11]. Perforation due to acute obstruction is usually reported in the cecum, with the occurrence of 3%–8% [12]. The cecum is particularly more susceptible to pressure‐induced perforation because of its larger diameter, thinner wall, and relatively limited blood supply. Several studies have reported perforation of sigmoid tumors following strangulation into the hernial sac. In an article conducted by Chern et al., 38 cases of colon cancer incarcerated in hernia were reported [13]. Sabra et al. reported that an 87‐year‐old man presented with typical signs of an incarcerated left inguinal area, which was found to have a perforated sigmoid tumor covered by hernia sac inside an inguinal hernia [14, 15]. Baldi et al. presented a rare case of sigmoid cancer incarcerated in a left inguinal hernia, infiltrating the spermatic cord [16]. In an interesting case reported by Toledo et al., cecal perforation occurred due to a closed‐loop obstruction in a patient with the adenocarcinoma of the sigmoid colon [17].

In the review of the literature, this is the first case report of cecal perforation following sigmoid tumor strangulation inside the inguinal hernia sac. In most published cases, colorectal cancers associated with inguinal hernias are typically sigmoid in origin and present as saccular or intrasaccular tumors incarcerated within the hernia sac, leading to localized obstruction or, less commonly, perforation at the site of the involved segment itself. However, in this patient, although a sigmoid adenocarcinoma was found to be strangulated within the left inguinal hernia sac, the site of perforation was unexpectedly located in the cecum, which is anatomically distant from the tumor. This suggests a different and indirect pathophysiological mechanism, most likely related to closed‐loop obstruction and markedly increased intraluminal pressure rather than direct tumoral invasion or localized ischemic perforation. The cecum, being the most vulnerable segment to pressure‐related perforation due to its larger diameter and thinner wall, became the site of rupture. Therefore, unlike previously reported cases where complications are confined to the herniated tumor‐bearing segment, this case uniquely demonstrates a remote colonic perforation secondary to a sigmoid tumor incarcerated within an inguinal hernia. To the best of our knowledge, such a combination, sigmoid carcinoma in an inguinal hernia with secondary cecal perforation, has been reported only very rarely in the literature. Total colectomy was performed due to cecal perforation and severe ischemic and distension‐related changes affecting the right colon secondary to prolonged obstruction. An end ileostomy was chosen because of the emergency setting and the patient's advanced age, which significantly increased the risk of primary anastomosis and postoperative complications.

## Author Contributions

**Mojtaba Ahmadinejad:** conceptualization, data curation, investigation, project administration, resources, supervision, validation, visualization. **Armin Tajik:** data curation, investigation, methodology, writing – original draft, writing – review and editing. **Hedieh Soltani:** methodology, writing – original draft, writing – review and editing. **Hadi Maleki:** data curation, investigation, methodology, supervision, validation, visualization. **Javad Zebarjadi Bagherpour:** conceptualization, data curation, methodology, supervision, validation, visualization, writing – review and editing.

## Funding

The authors have nothing to report.

## Disclosure

*Declaration of generative AI in scientific writing*: The authors declare that they did not use generative artificial intelligence (AI) in the scientific writing of this manuscript.

## Ethics Statement

Ethical approval for this study was provided by the Ethical Committee Alborz University of Medical Sciences Research Committee.

## Consent

Written informed consent was obtained from the patient for publication of this case report and accompanying images. A copy of the written consent is available for review by the Editor‐in‐Chief of this journal on request.

## Conflicts of Interest

The authors declare no conflicts of interest.

## Data Availability

The datasets used and/or analyzed during the current study are available from the corresponding author on request, with the approval of the patient.
